# Continuous AMD3100 Treatment Worsens Renal Fibrosis through Regulation of Bone Marrow Derived Pro-Angiogenic Cells Homing and T-Cell-Related Inflammation

**DOI:** 10.1371/journal.pone.0149926

**Published:** 2016-02-22

**Authors:** Juan Yang, Fengming Zhu, Xiaohui Wang, Weiqi Yao, Meng Wang, Guangchang Pei, Zhizhi Hu, Yujiao Guo, Zhi Zhao, Pengge Wang, Jingyi Mou, Jie Sun, Rui Zeng, Gang Xu, Wenhui Liao, Ying Yao

**Affiliations:** 1 Division of Nephrology, Tongji Hospital, Tongji Medical College, Huazhong University of Science and Technology, 1095 Jiefang Ave, Wuhan 430030, Hubei, China; 2 Department of Nephrology, Fifth Hospital of Wuhan, 122 Xianzheng Street, Hanyang district, Wuhan 430050, Hubei, China; 3 Wuhan Hamilton Biotechnology-Co.LTD., B6-4, Wuhan institute of biotechnology, #666 Gaoxin Road, Wuhan 430073, Hubei, China; 4 Department of Geriatrics, Tongji Hospital, Tongji Medical College, Huazhong University of Science and Technology, 1095 Jiefang Ave, Wuhan 430030, Hubei, China; Medical College of Wisconsin, UNITED STATES

## Abstract

AMD3100 is a small molecule inhibitor of chemokine receptor type 4 (CXCR4), which is located in the cell membranes of CD34+ cells and a variety of inflammatory cells and has been reported to reduce organ fibrosis in the lung, liver and myocardium. However, the effect of AMD3100 on renal fibrosis is unknown. This study investigated the impact of AMD3100 on renal fibrosis. C57bl/6 mice were subjected to unilateral ureteral obstruction (UUO) surgery with or without AMD3100 administration. Tubular injury, collagen deposition and fibrosis were detected and analyzed by histological staining, immunocytochemistry and Western Blot. Bone marrow derived pro-angiogenic cells (CD45+, CD34+ and CD309+ cells) and capillary density (CD31+) were measured by flow cytometry (FACS) and immunofluorescence (IF). Inflammatory cells, chemotactic factors and T cell proliferation were characterized. We found that AMD3100 treatment did not alleviate renal fibrosis but, rather, increased tissue damage and renal fibrosis. Continuous AMD3100 administration did not improve bone marrow derived pro-angiogenic cells mobilization but, rather, inhibited the migration of bone marrow derived pro-angiogenic cells into the fibrotic kidney. Additionally, T cell infiltration was significantly increased in AMD3100-treated kidneys compared to un-treated kidneys. Thus, treatment of UUO mice with AMD3100 led to an increase in T cell infiltration, suggesting that AMD3100 aggravated renal fibrosis.

## Introduction

Renal fibrosis is the final common pathway of chronic kidney disease, and it ultimately leads to end stage renal disease, which requires sustained drug administration or renal replacement therapy. As such, renal fibrosis is an increasing global health problem, and efficient treatments are needed [1–3]. Many studies have focused on inhibiting myofibroblast activation and proliferation [4–6]; however, these treatment strategies require a long time to achieve good outcomes. Thus, simple and convenient therapeutic strategies for renal fibrosis are urgently needed.

Peritubular microvascular rarefaction and impaired angiogenesis are early fibrotic events that have long been considered to be important in the pathomechanism of the initiation of renal fibrosis in CKD [7]. Thus, angiogenesis is a potential target for the treatment of renal fibrosis [8]. However, the quanity of resident cells which give rise to the functional vasculature in kidney is very limited [9, 10], the majority of them are from bone marrow [11–13]. Cells mobilized from bone marrow into peripheral circulation that participate vascular repair and angiogenisis, which was originally named as endothelial progenitor cells (EPCs) [14], but now it was proved these cells actually was pro-angiogenic cells [15]. Therefore, mobilizing bone marrow derived pro-angiogenic cells into peripheral blood and injured kidneys plays a key role in promoting new blood vessel growth in the kidneys.

Migration of pro-angiogenic cells from bone marrow (BM) is highly dependent on the chemokine stromal cell–derived factor-1 α (SDF-1) and its receptor, CXCR4 [16]. SDF-1 binds to CXCR4 on precursor cells leading to retention of hematopoietic stem cells in the BM. Degradation of the SDF-1 concentration gradient in the BM and increased expression of CXCR4 on precursor cells [17] causes bone marrow precursor cells to be recruited to injured tissues [18]. AMD3100 is a small molecule inhibitor of CXCR4 that interferes with SDF-1/CXCR4-mediated BM retention of precursor of pro-angiogenic cells, resulting in mobilization of pro-angiogenic cells into the blood [19], migration of pro-angiogenic cells into target organs [20, 21], and alleviation of tissue injury. However, those reports were principally focused on fibrosis in the liver, lungs and myocardium [22–24]. The effect of AMD3100 on renal fibrosis is still unknown: whether AMD3100 treatment can accelerate the mobilization of bone marrow derived pro-angiogenic cells, increase renal angiogenesis and alleviate renal fibrosis requires extensive investigation.

In contrast to our expectations, the present study showed that AMD3100 does not increase renal angiogenesis or attenuate renal fibrosis; instead, it worsens UUO-induced renal fibrosis by exacerbating T cell-related renal inflammation.

## Materials and Methods

### Animals

Male C57bl/6 mice (6–7 weeks old, weighing 20 g) were purchased from the Beijing Hua Fukang Laboratory Animal Technology Co., Ltd, Beijing, China. The animals were housed at Tongji Medical College Animal Care Unit. The animals were acclimated to the housing environment, which was SPF and had a temperature of 22°C and a 12h/12h light/dark cycle for a week. Then, they were randomly divided into following experimental groups, with 8 mice in each group: normal (no specific intervention), UUO+AMD3100 (mice received UUO surgery and 2 mg/kg AMD3100), and UUO+PBS (mice received UUO surgery and the same volume of PBS). AMD3100 and PBS were administered via intraperitoneal injection every day until sacrifice. The UUO surgery was performed as previously reported [25]. Briefly, animals were anesthetized using 1% sodium pentobarbital (0.008 mL/g) and were placed on a heated surgical pad. The left ureter was visualized via a flank incision and was double ligated. After surgery, the general health of the animals was monitored daily by the investigators and/or by members of the clinical veterinary staff to detect any signs of discomfort. No deaths occurred during the surgery or the subsequent observation period. On the fifth day after surgery, the animals were euthanized by cervical dislocation, and their left kidneys were harvested for analysis. All reasonable measures were taken to minimize suffering and ensure the health and well-being of the animals during the course of the study. All experimental procedures were conducted in accordance with NIH guidelines and were approved by the Animal Care and Use Committee (ACUC) of Tongji Hospital.

### Histologic, immunocytochemical and immunofluorescence staining

Paraffin-embedded renal sections (3 μm) were subjected to periodic Acid-Schiff staining (PAS) to evaluate tubular injury, Masson trichrome (Masson) and Sirius red staining to estimate renal interstitial fibrosis level. The renal tubular damage score was based on tubular necrosis grade, cast formation, tubular dilation, and brush border loss, with scores corresponding to the following percentages of renal tubular damage: 0, 0%; 1, ≤10%; 2, 11 to 25%; 3, 26 to 45%; 4, 46 to 75%; and 5, ≥76%. For IHC, renal sections (3 μm) were incubated with 3% H_2_O_2_ for 10 min, and then, for immunocytochemical and immunofluorescence staining, they were incubated with BSA for 30 min at room temperature. After being washed, the slides were incubated with primary antibodies against either α-SMA (1:100, Abcam), PDGFR-β (1:100, Epitomics), collagen-IV (1:100, Abcam), CD31 (1:100, BD), CD3 (1:100, Abcam), or PCNA (1:50, Santa Cruz) at 4°C overnight. The slides were then incubated with the appropriate secondary antibody (HRP-conjugated secondary antibody for IHC and fluorescent labeled secondary antibody for IF). For double staining, the slides were incubated with two kinds of primary antibody and secondary antibody simultaneously.

Ten randomly selected non-overlapping fields were captured for each slide and were analyzed using Image Pro Plus software (Media Cybernetics, Rockville, MD, USA) in a blinded manner by two pathologists.

### Western Blot

Renal tissues were homogenized in RIPA lysis buffer containing a protease inhibitor cocktail. Total protein concentrations were determined using a BCA assay kit following the manufacturer’s instructions. Fifty micrograms of protein was separated by SDS-PAGE, and the separated proteins were transferred to PVDF membranes. The membranes were blocked with 5% nonfat milk in TBS with 0.1% Tween-20 for 1 h at 37°C and then probed with antibodies against α-SMA (1:2000, Abcam), PDGFR-β (1:2000, Epitomics), GAPDH (1:3000, Minipore), or HIF-1α (1:1000, Abcam) at 4°C overnight. After being washed, the blots were incubated with an HRP-conjugated anti-IgG, and the target bands were visualized with ECL plus reagents following the manufacturer’s instructions. The intensities of the target bands were analyzed by densitometry and were normalized to GAPDH or β-actin using Quantity One software (BioRad, CA, USA); prior to analysis, the intensities of the target bands were normalized to the corresponding values of the control group.

### Real-time quantitative RT-PCR

Total RNA extraction and reverse transcription were conducted using the GoScript reverse transcription system (Promega, USA). PCR enzymes & master mixes (Thermo Scientific, USA) were used for real-time PCR with primers specific for mouse GAPDH, HIF-1α, TNF-α, IL-6, IL-10, IFN-γ, CCL4, CCL5, CX3CL1, CXCL10 and CXCL9. The sequences for all primers are shown in Table 1. Relative expression levels were normalized to GAPDH and calculated using the 2^−ΔΔCt^ method.

**Table 1:** Primer sequences.

	Forward	Reverse
Mouse GAPDH	5’-TCAACGATTTGGTCGTATT-3’	5’-CTGTGGTCATGAGTCCTTCC-3’
Mouse HIF-1α	5’-GATGACGGCGACATGGTTTAC-3’	5’-CTCACTGGGCCATTTCTGTGT-3’
Mouse TNF-α	5’-CCCTCACACTCAGATCATCTTCT-3’	5’-GCTACGACGTGGGCTACAG-3’
Mouse IL-6	5’-GAGGATACCACTCCCAACAGACC-3’	5’-AAGTGCATCATCGTTGTTCATACA-3’
Mouse IL-10	5’-CCAAGTGCTGCCGTCATTTTC-3’	5’-TCCCTATGGCCCTCATTCTCA-3’
Mouse IFN-γ	5’-ACAGCAAGGCGAAAAAGGATG-3’	5’-TGGTGGACCACTCGGATGA-3’
Mouse CCL4	5’-TTCCTGCTGTTTCTCTTACACCT-3’	5’-CTGTCTGCCTCTTTTGGTCAG-3’
Mouse CCL5	5’-GCTGCTTTGCCTACCTCTCC-3’	5’-TCGAGTGACAAACACGACTGC-3’
Mouse CX3CL1	5’-CTGCCCTCACTAAAAATGGTGG-3’	5’-AATGTGGCGGATTCAGGCTT-3’
Mouse CXCL9	5’-TCCTTTTGGGCATCATCTTCC-3’	5’-TTTGTAGTGGATCGTGCCTCG-3’
Mouse CXCL10	5’-CCAAGTGCTGCCGTCATTTTC-3’	5’-TCCCTATGGCCCTCATTCTCA-3’

### Flow cytometry

Cells from bone marrow, peripheral blood and the kidney were collected and diluted into single cell suspensions. Briefly, the femur and tibia were cut out, and the muscles were removed. Then, bone marrow cells were collected by flushing the shafts of the bones with PBS using a 10 mL syringe and a 26-gauge needle. Then, the red blood cells were lysed, and the remaining cells were passed through 30 μm nylon mesh. For kidney tissue preparation, the whole kidney was diced and incubated with Liberase (0.5 mg/mL; Roche Applied Science, Indianapolis) at 37°C for 1 hour; then, the cell clumps were removed by passing the cells through a 40 μm filter. Finally, 1×10^6^ cells from different sources were resuspended in 100μL PBS-BSA (1%wt/vol) and were incubated with different primary antibodies for 40 min at room temperature. The following antibodies were used: FITC-conjunctive CD45 (Ebioscience), APC-conjunctive CD34 (Biolegend), PE-conjunctive CD309 (Biolegend), FITC-conjunctive CD3 (Ebioscience), FITC-conjunctive CD4 (Ebioscience), FITC-conjunctive CD11b (Ebioscience), and FITC-conjunctive F4/80 (Ebioscience). The percentages and numbers of positive cells were determined by BD FACS.

### Statistical analysis

All data are expressed as the means±S.D. Comparisons between two groups were accomplished using the unpaired t-test or nonparametric Mann-Whitney test. All data were analyzed using SPSS 18.0 software for Windows. *p*<0.05 was considered to indicate a statistically significant difference.

## Results

### AMD3100 aggravated UUO-induced tubulointerstitial damage and fibrosis

**Fig 1:**
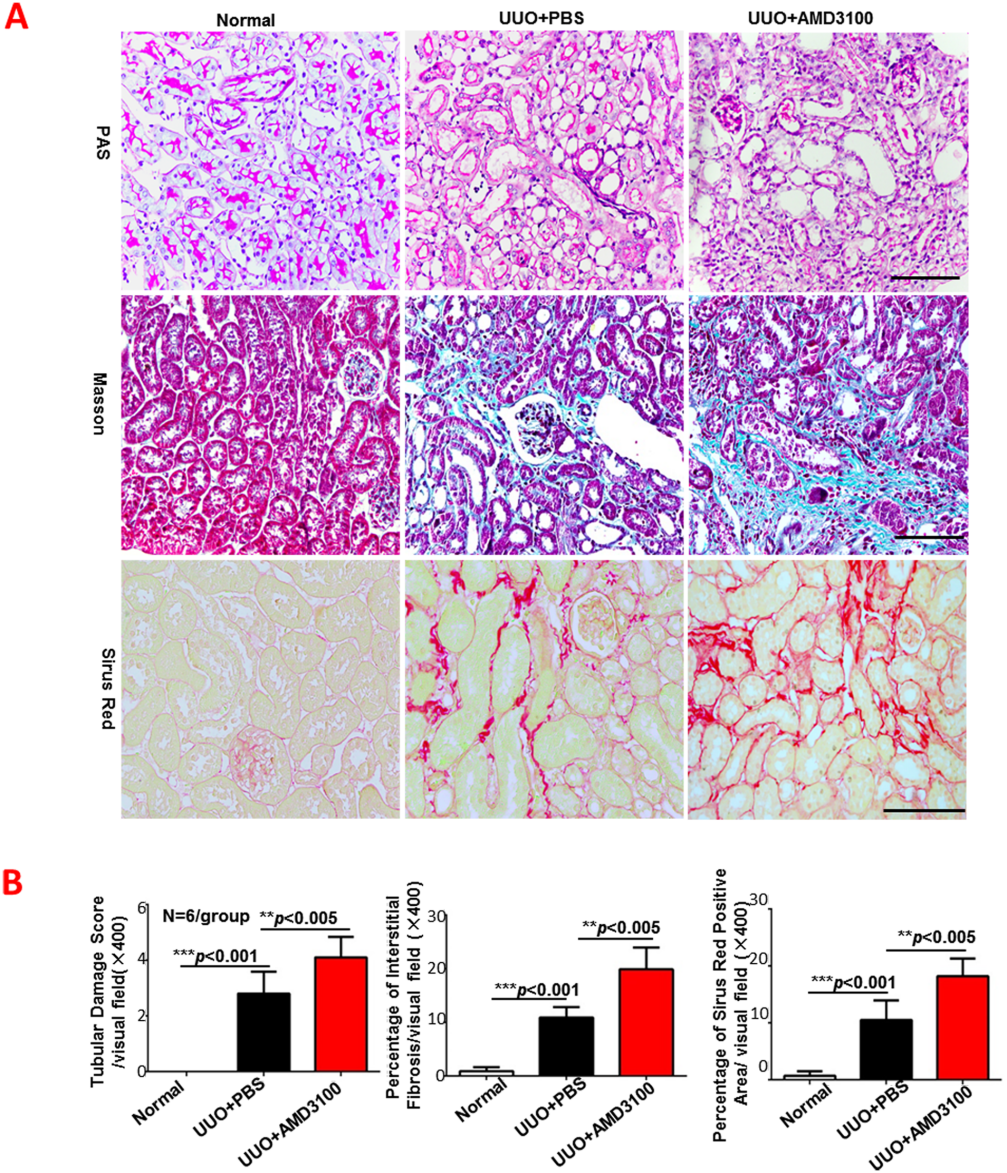
AMD3100 aggravated UUO-induced tubulointerstitial damage and fibrosis. A. Paraffin-embedded renal sections (3 μm) were subjected to Periodic Acid-Schiff staining (PAS), Masson trichrome staining and Sirius red staining. B. Tubular damage score was determined based on PAS staining; renal interstitial fibrosis was evaluated by Masson staining; and the areas positive for Sirius red staining were also analyzed. (***p<0.001, **p<0.005, scale = 100μm, magnification×400)

Many studies have shown that administration of AMD3100 reduces tissue damage in fibrotic lung, liver and myocardium and in cell lines [22, 26, 27]. In this study, we examined the morphology changes and collagen deposition in UUO-induced renal fibrosis with and without AMD3100 treatment. As shown in Fig 1, the renal pathological changes caused by UUO surgery (tubular necrosis, cast formation, tubular dilation, and brush border loss) were worse in the UUO+AMD3100 group than in the control group, which did not meet with our expectations. The renal tubular damage score in the UUO+AMD3100 group was much higher (mean 4.1 vs. 2.8, *p<*0.005) than that in the UUO+PBS group. In support of that finding, interstitial collagen deposition, which was evaluated by Masson trichrome staining and Sirius red staining, was significantly increased in the UUO+AMD3100 group compared to the UUO+PBS group by approximately 1.8-fold (19.9 vs.10.9, *p<*0.005 for Masson trichrome; 18.2 vs. 10.5, *p<*0.005 for Sirius red). However, no further deterioration renal function was found in UUO+AMD3100 group, which may due to the compensatory function of contralateral normal kidney (Part A in S1 Fig). Taken together, our data indicate that AMD3100 treatment does not alleviate fibrosis but, rather, increases tubulointerstitial damage and renal fibrosis.

### AMD3100 treatment increased α-SMA, PDGFR-β and collagen-IV expression

**Fig 2:**
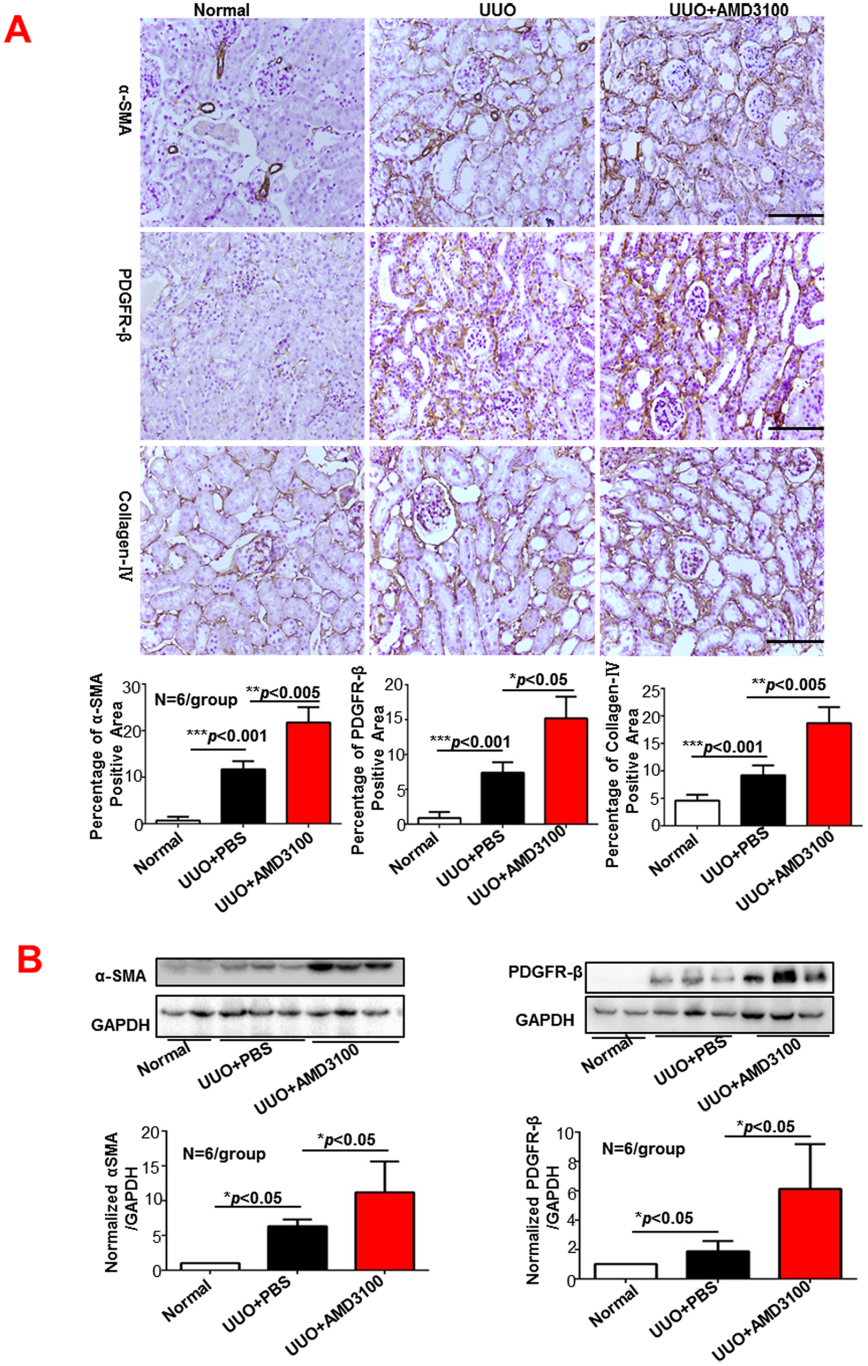
AMD3100 increased α-SMA, PDGFR-β and collagen-IV expression levels following UUO. A. Immunohistochemistry (magnification×400). B. Western Blot (magnification×400). (***p<0.001, **p<0.005, *p<0.05, scale = 100μm)

We also detected the effect of AMD3100 on the expression of the specific fibrosis markers α-SMA, PDGFR-β and collagen-IV by immunohistochemistry (IHC) and Western Blot. As shown in Fig 2A, the IHC analysis showed that the AMD3100-treated kidneys had more intense and more widely distributed expression of α-SMA (1.85-fold more than the PBS group, *p*<0.005), PDGFR-β (2.05-fold more than the PBS group, *p*<0.05) and collagen-IV (2.03-fold more than the PBS group, *p*<0.005) than the PBS-treated kidneys. The Western Blot findings for α-SMA and PDGFR-β were consistent with the IHC data (Fig 2B), indicating that AMD3100 treatment promoted the activation of myofibroblasts and increased renal interstitial fibrosis.

### AMD3100 inhibited bone marrow derived pro-angiogenic cells migration but did not alter UUO-induced vascular rarefaction or tissue hypoxia

**Fig 3:**
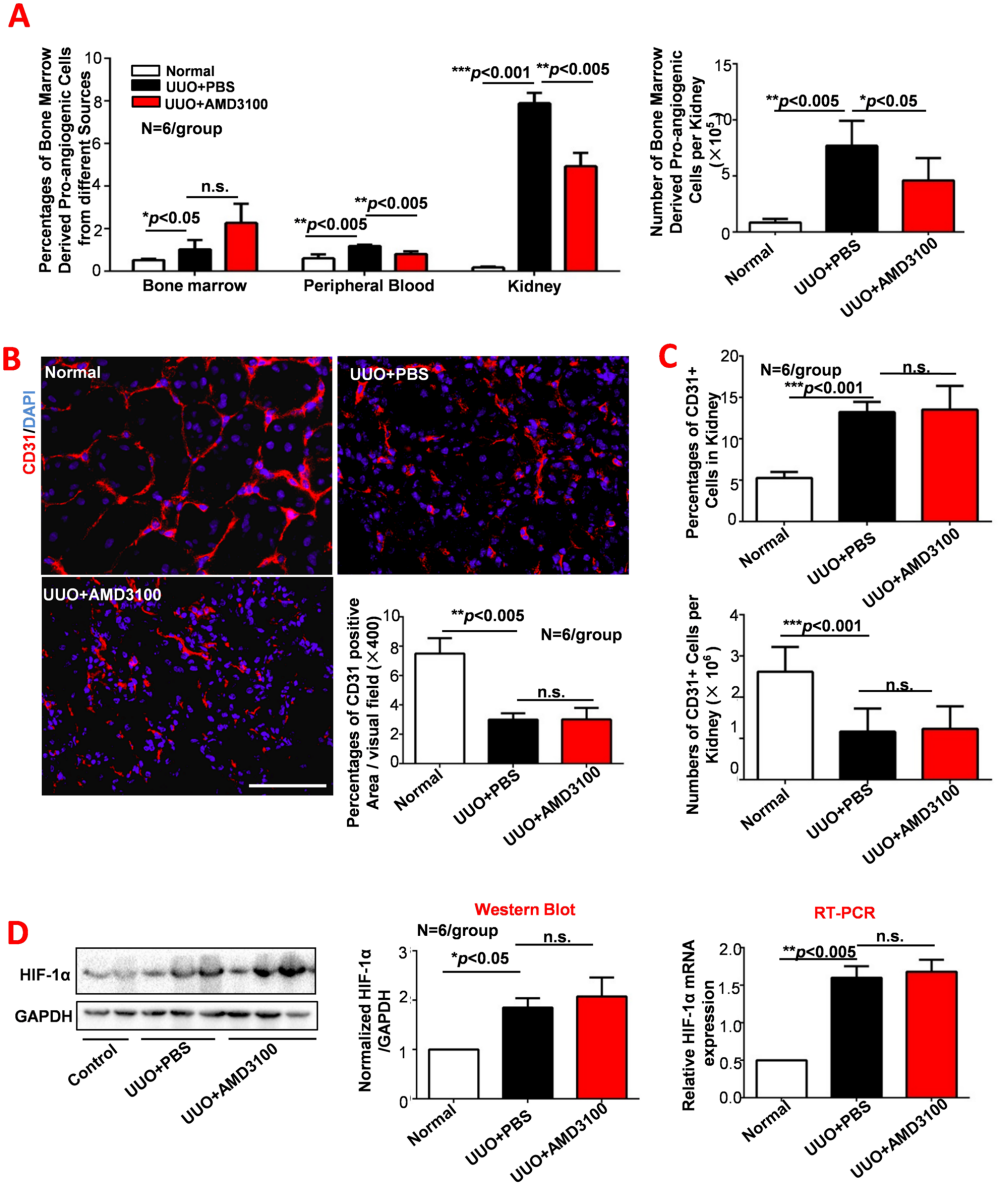
AMD3100 inhibited bone marrow derived pro-angiogenic cells migration but did not alter UUO-induced vascular rarefaction or tissue hypoxia. A. Percentages of bone marrow derived pro-angiogenic cells in different tissues and the number of bone marrow derived pro-angiogenic cells in the kidneys were analyzed by flow cytometry. B. CD31 expression was detected by immunofluorescence. C. The percentage and number of CD31-positive cells in the kidney were determined by flow cytometry. D. The expression of HIF-1α was detected by Western Blot and RT-PCR. (***p<0.001, **p<0.005, *p<0.05, scale = 100μm, magnification×400)

Angiogenesis is a potential target for the treatment of renal fibrosis because bone marrow derived pro-angiogenic cells that migrate into injured kidneys are thought to give rise to functional vasculature and to alleviate renal fibrosis. Therefore, we hypothesized that AMD3100, an activator of bone marrow derived pro-angiogenic cells mobilization, would inhibit renal fibrosis by promoting these cells mobilization and migration into the kidneys. However, our data showed that the percentages of bone marrow derived pro-angiogenic cells in the peripheral blood and kidneys of the UUO+AMD3100 group were much lower than those in the UUO+PBS group (*p*<0.005), indicating that AMD3100 inhibited UUO-induced mobilization and migration of bone marrow derived pro-angiogenic cells. To exclude the impact of total parenchymal cell loss during the development of UUO, the numbers of bone marrow derived pro-angiogenic cells in the kidneys was determined. As shown in Fig 3A (right panel), the bone marrow derived pro-angiogenic cells number in the kidneys of the UUO+AMD3100 group was decreased by almost half compared to that of the UUO+PBS group (*p*<0.05). These data are consistent with those reported by Kentaro Jujo [24], who found that continuous administration of AMD3100 inhibited bone marrow derived pro-angiogenic cells migration.

As the main functions of bone marrow derived pro-angiogenic cells are vascular repair and angiogenesis in response to tissue injury, capillary density (CD31 positivity) was determined in the three groups by IF and FACS (Fig 3B and 3C). Remarkable capillary loss was observed in both the UUO+PBS and UUO+AMD3100 groups (*p*<0.005), with no statistically difference between them (*p>*0.05). Similarly, no difference was observed in the percentage or number of CD31-positive cells in the kidney of the UUO+PBS and UUO+AMD3100 groups (both *p*>0.05), suggesting that the inhibition of bone marrow derived pro-angiogenic cells migration by AMD3100 does not decrease renal vascular density. The above results inspired us to examine renal hypoxia by detecting the expression of HIF-1α, a marker of tissue hypoxia. As expected, no difference in hypoxia was observed between the UUO+AMD3100 and UUO+PBS groups (Fig 3D). These data suggests that AMD3100 inhibited the migration of bone marrow derived pro-angiogenic cells did not induce capillary loss or tissue hypoxia. Additionally, decreased bone marrow derived pro-angiogenic cells induced by AMD3100 administration also didn’t impact the process of Endothelial-to-Mesenchymal Transition (Part B in S1 Fig).

### Continuous AMD3100 treatment increased inflammatory cytokine secretion and promoted CD3-positive cell infiltration

**Fig 4:**
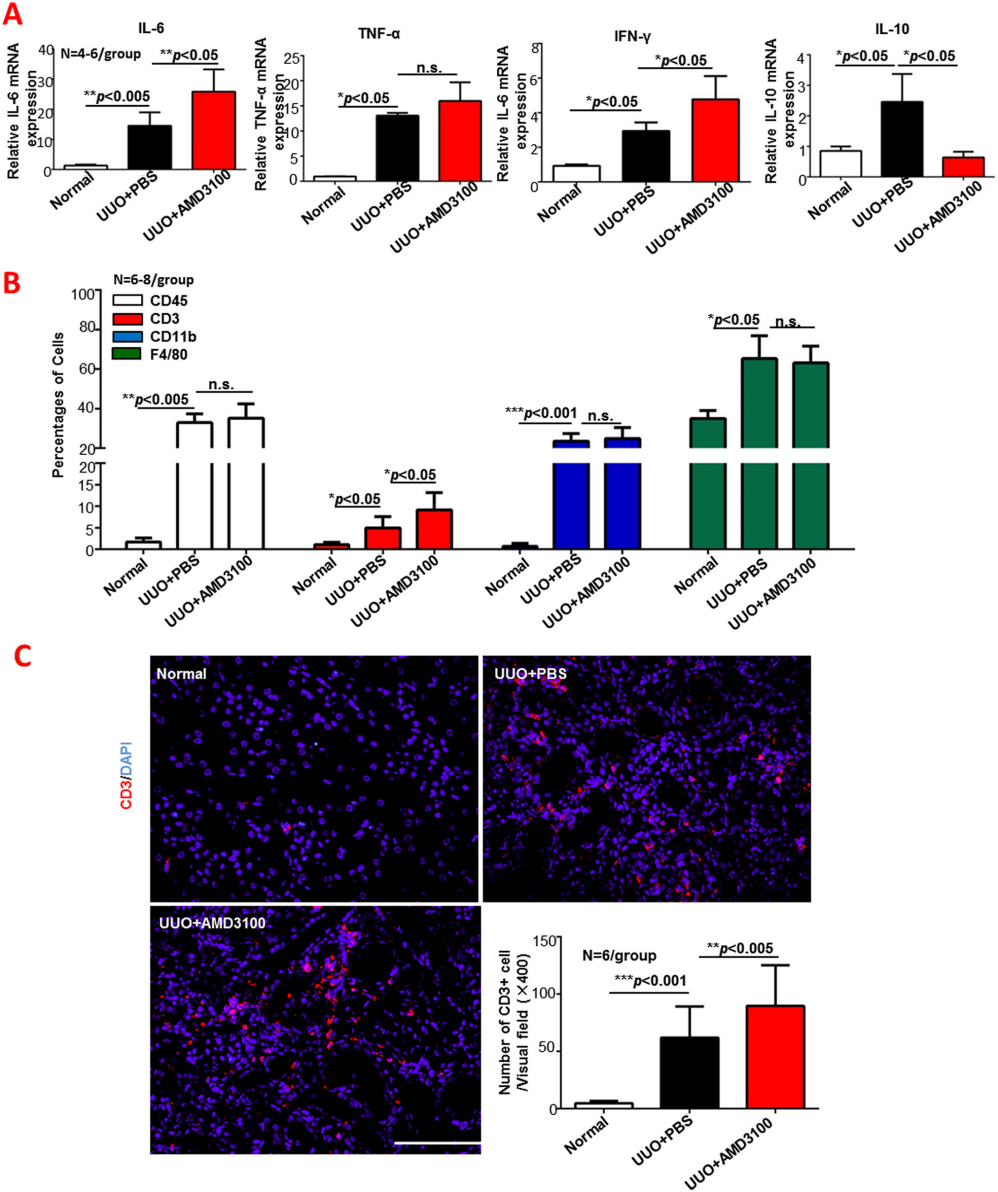
AMD3100 increased inflammatory cytokine secretion, and promoted CD3-positive cell infiltration following UUO. A. Expression of renal inflammatory cytokines (IL-6, TNF-α, IL-10 and IFN-γ) was detected by RT-PCR. B. Percentages of different inflammatory cell types (cells positive for CD45, CD3, CD11b and F4/80) in the kidney were measured by flow cytometry. C. Immunofluorescence was used to further determine CD3-positive cell infiltration into the kidney, and the number of infiltrated CD3-positive cells was determined. (***p<0.001, **p<0.005, *p<0.05, n.s. means no significant difference, scale = 100μm, magnification×400)

Bone marrow derived pro-angiogenic cells are known to be involved in vascular repair, but recent evidence shows that they also play an important role in the anti-inflammatory response when the local microenvironment is not conducive to vascular repair [11]. Our observation of the inhibition of bone marrow derived pro-angiogenic cells migration into UUO kidneys by AMD3100 inspired us to perform experiments on AMD3100-treated kidneys in the presence of the inflammatory response. The inflammatory cytokines IL-6, TNF-α, IL-10 and IFN-γ were detected by RT-PCR (Fig 4A). After AMD3100 treatment, the mRNA expressions of IL-6 and IFN-γ were increased (both *p*<0.05), while the mRNA expression of IL-10 was significantly decreased (*p*<0.05). AMD3100 also up-regulated TNF-α expression, but the difference was not statistically significant (*p*>0.05). To investigate what inflammatory cell types were influenced by AMD3100, the percentages of total leukocytes (CD45+), T cells (CD3+), and macrophages (CD11b or F4/80) were measured by FACS. As shown in Fig 4B, CD3+ cells were obviously increased by AMD3100 administration (almost 2-fold more than that of the PBS group, *p*<0.05). The immunofluorescence staining results (Fig 4C) were consistent with the FACS data: the number of CD3+ cells/visual field in the UUO+AMD3100 group was much greater than that in the UUO+PBS group (62.05 vs. 98.7, *p*<0.005). In summary, AMD3100 administration to UUO mice exacerbated renal interstitial T cell infiltration, resulting in increased production of the pro-inflammatory cytokines IL-6 and IFN-γ and decreased expression of the anti-inflammatory cytokine IL-10.

### Continuous AMD3100 treatment increased renal T cell chemotaxis but not local proliferation

**Fig 5:**
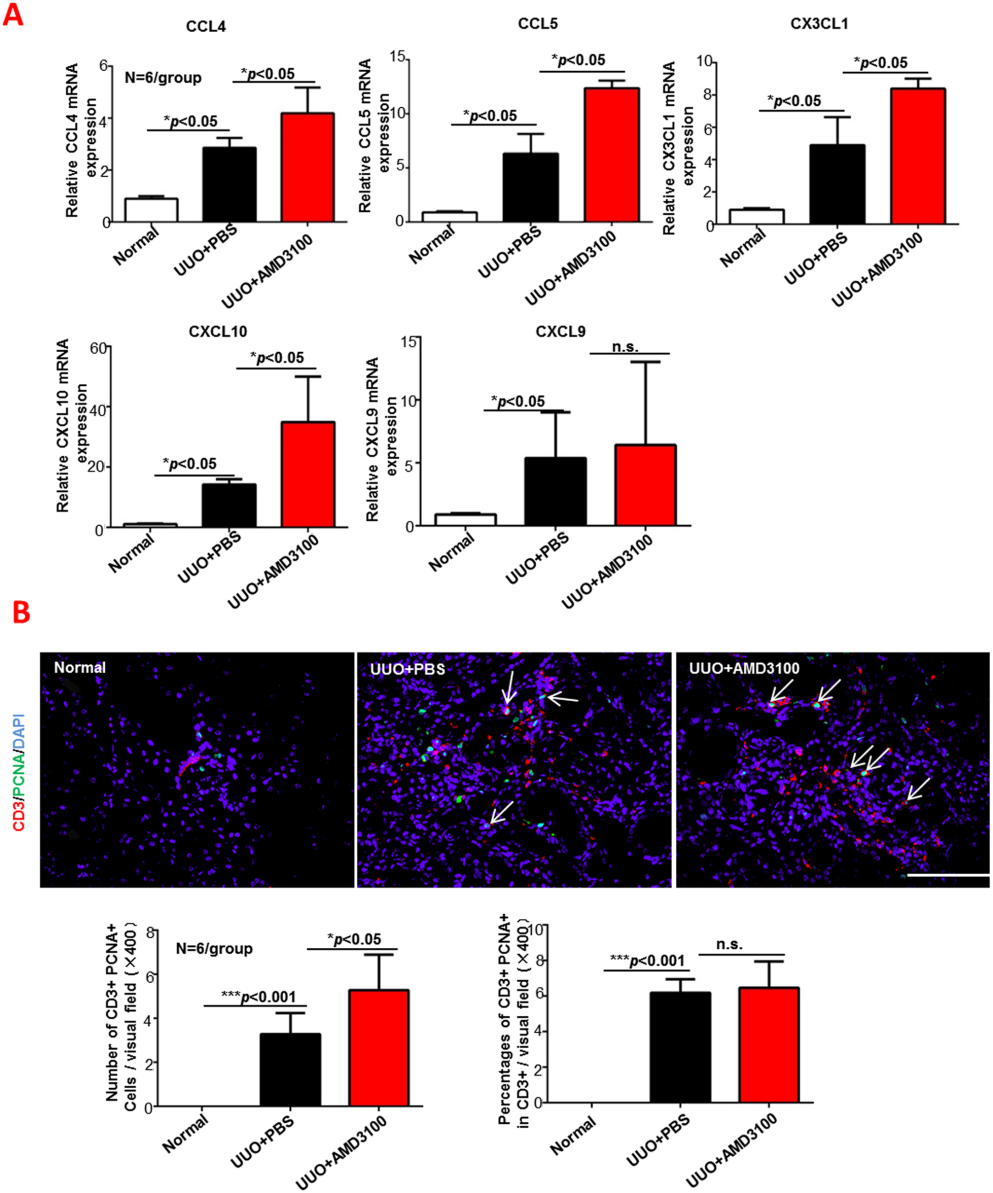
AMD3100 promoted T cell chemotaxis but not proliferation. A. Fold changes in T cell-related chemokines were measured by RT-PCR. B. Double staining of CD3 (red) and PCNA (green) was examined by immunofluorescence, and the number of double-positive cells (white arrows) was analyzed. (***p<0.001, **p<0.005, *p<0.05, n.s. means no significant difference, scale = 100μm, magnification×400)

Because CD3+ T cells were increased in the UUO+AMD3100 group, we attempted to determine whether this increase was caused by T cell migration from the peripheral blood or by local T cell proliferation. RT-PCR was adopted to detect T cell-related chemokines (CCL4, CCL5, CX3CL1, CXCL10 and CXCL9), and double staining of CD3 (red) and PCNA (green) was examined by immunofluorescence. As shown in Fig 5A, all chemokines, except for CXCL9, was increased by AMD3100 administration as follows: CCL4 (1.5-fold more than the PBS group, *p*<0.05), CCL5 (2-fold more than the PBS group, *p*<0.05), CX3CL1 (1.7-fold more than the PBS group, *p*<0.05) and CXCL10 (3.1-fold more than the PBS group, *p*<0.05). Interestingly, immunofluorescence (Fig 5B) verified that the number of local T cells was increased by AMD3100 administration (3.28 without AMD3100 vs. 5.28 with AMD3100, *p*<0.05), but the proportion of proliferating T cells was not increased by AMD3100 administration (6.18 in PBS vs. 6.46 in AMD3100 group, *p*>0.05). Considering all of these results collectively, the strong infiltration of CD3+ T cells into AMD3100-treated kidneys was induced by a T cell chemotaxis response to increased release of T cell chemokines, not to local T cell proliferation.

## Discussion

Unlike most previous reports, this study provided evidence of an unexpected pro-fibrotic effect of AMD3100 in animal model of UUO-induced early renal fibrosis. The principle histomorphometric findings were that AMD3100 aggravated renal damage, activated myofibroblast and increased collagen deposition. The mechanism of AMD3100-induced renal fibrosis was related to decreased migration of bone marrow derived pro-angiogenic cells and enhanced T cell infiltration. Given that AMD3100 has been used for certain diseases in clinical practice, our study suggests the need for more cautious use of AMD3100 in patients with renal disease.

Bone marrow derived pro-angiogenic cells are endothelial precursor cells involved in vascular repair and neovascularization after tissue injury. Accumulated evidence [11, 28, 29] has demonstrated that pro-angiogenic cells transplantation is a new therapy for fibrotic diseases that acts by ameliorating vascular damage and rarefaction associated with renal fibrosis. Another highly effective method for increasing the number of local pro-angiogenic cells is mobilization of it from bone marrow into other tissues, and this has been shown to be controlled by the SDF-1/CXCR4 system [16, 30].

AMD3100, also called plerixafor and Mozobil^®^, is a CXCR4 inhibitor that mobilizes bone marrow derived pro-angiogenic cells by interfering with SDF-1/CXCR4 mediated bone marrow retention of pro-angiogenic cells [31, 32]. However, our study showed that administration of AMD3100 inhibited the homing of bone marrow derived pro-angiogenic cells and deteriorated renal damage and fibrosis, which is consistent with some previous reports. Isabelle Petit [30] et al. reported that chronic AMD3100 treatment inhibited bone marrow derived pro-angiogenic cells mobilization and migration and aggravated fibrosis in animals with myocardial infarction [33]. Another paper compared the impacts of a single dose and continuous infusion of AMD3100 on myocardial infarction and showed that continuous infusion aggravated cardiac capillary loss and fibrosis [24]. Interestingly, delivering AMD3100 at different times has different effects [34]. Therefore, we hypothesized that the decrease in bone marrow derived pro-angiogenic cells homing and the increase in fibrosis with AMD3100 treatment observed in our study may be because AMD3100 was administered continuously (daily from day zero). The decline in bone marrow derived pro-angiogenic cells homing observed with continuous AMD3100 treatment was likely due to prolonged disruption of SDF-1–CXCR4 binding.

A recent study by Hohenstein and colleagues suggested that extrarenal endogenous endothelial progenitor cells (EPCs) do not home to the kidney at all [35], which seems not consistent with our observations. In fact, the detected endothelial progenitor cells were ECFC (CD34+CD309+CD133-CD45-) which is defined based on the cell culture assay and cell colony forming assay in vitro. And what we detected is bone marrow derived pro-angiogenic cells (CD45+CD34+CD309+); we didn’t further investigate whether it is ECFC or EC-CFU. So we just focused on different pro-angiogenic cells subsets. As Hohenstein mentioned in his study, the definition of so-called EPCs was questionable, maybe the term PACs (pro-angiogenic cells) is better to describe this kind of cells, which is also proposed and supported by Basile and Yoder [15], and was just used in this study.

Our data indicate that decreased bone marrow derived pro-angiogenic cells migration did not increase capillary loss or tissue hypoxia; however, it did increase inflammatory cytokine secretion and renal damage. Previous studies have shown that bone marrow derived pro-angiogenic cells not only directly contribute to the functional endothelium and support resident endothelial cells,but also influence surrounding parenchymal cells via paracrine mechanisms, including the release of growth factors and microvesicles (MVs) [36, 37]. Pro-angiogenic cells infusion not only attenuated vascular rarefaction but also reduced tissue damage and inflammatory cell infiltration, which explains why the decreased number of bone marrow derived pro-angiogenic cells in the UUO kidney was accompanied by increased renal damage in the present study. We propose two possible explanations for the lack of further reduction in vascular density to that observed in the control group in the presence of AMD3100. The first is the limitation of the animal model: unilateral ureteral ligation is designed to be a continuous progressive fibrotic process, and the micro-environment generated by that procedure is not suitable for bone marrow derived pro-angiogenic cells to perform endothelial repair; thus, the paracrine effects of bone marrow derived pro-angiogenic cells in this scenario may be focused on renal parenchymal cell repair. The other possible explanation is that the promotion of mobilization and migration of other stem cells (such as MSC sand HSCs) by AMD3100 may have counteracted the adverse effects caused by bone marrow derived pro-angiogenic cells reduction.

The shift in the T cell cytokine secretion profile in AMD3100-treated kidneys is another explanation for the increased renal damage observed in the presence of AMD3100 in this study. AMD3100 treatment promoted IL-6 and IFN-γ expression and impaired IL-10 secretion. IL-10 is necessary for AMD3100-induced bone marrow derived pro-angiogenic cells mobilization [38]. Deficiency in IL-10 impairs the survival of bone marrow-derived pro-angiogenic cells and aggravates fibrosis [39], which is consistent with the observation of the changes in the levels of bone marrow derived pro-angiogenic cells and T cells in this study.

CXCR4 is highly expressed on T cells [40]. AMD3100 was previously shown to redistribute T cells from the bone marrow and thymus to the blood and peripheral tissues in mice [41, 42]. Administration of AMD3100 led to increased CD4+ and/or CD8+ T cell infiltration [41, 43] and decreased the quantity of Tregs [44] in peripheral organs, which is in accordance with our finding that renal T cell infiltration was increased after AMD3100 administration. We also demonstrated that increased renal T cell chemotaxis is another mechanism of AMD3100-induced T cell infiltration.

Additionally, we verified that this enhanced infiltration of CD3 T cells was due to increased mobilization and migration of T cells rather than local proliferation of T cells. As increased T cell infiltration is a strong pro-fibrotic factor in renal fibrosis [25, 45], we propose that the increase in T cells and the decrease in bone marrow derived pro-angiogenic cells in the kidneys caused by AMD3100 led to the observed increase in renal fibrosis.

There are still some questions regarding the effects of AMD3100 that require further explanation. 1) Does a single dose of AMD3100 attenuate renal fibrosis as reported in other organs? 2) What specific T cell subtype is affected by AMD3100? 3) Is there cross-talk regarding the changes in bone marrow derived pro-angiogenic cells and T cells induced by AMD3100? We will answer these questions by performing additional studies in the future.

In summary, the current study indicated that continuous AMD3100 treatment worsens renal fibrosis by regulating bone marrow derived pro-angiogenic cells and T cell infiltration into UUO kidneys. This suggests that in clinical practice, the adverse effect of AMD3100 on renal fibrosis must be given serious consideration.

## Supporting Information

S1 Fig Effects of AMD3100 on renal function and Endothelial-to-Mesenchymal Transition. A. The effect of AMD3100 on renal function. Blood Urea Nitrogen (BUN) of three groups were detected and analyzed. B. The changes of Endothelial-to-Mesenchymal Transition after AMD3100 administration. Double staining of CD31 (red) and α-SMA (green) was detected by immunofluorescence, and the number of co-positive vascular (yellow area) was analyzed. Lower right corner pictures in IF figures were the higher magnification of inset areas with white border. (***p<0.001, **p<0.005, *p<0.05, n.s. means no significant statistical difference, scale = 100μm, magnification×400)
(TIF)
