# Supplementary figures and images for: Continuous AMD3100 Treatment Worsens Renal Fibrosis through Regulation of Bone Marrow Derived Pro-Angiogenic Cells Homing and T-Cell-Related Inflammation
Source: PLoS One. 2016 Feb 22;11(2):e0149926. doi: 10.1371/journal.pone.0149926 (PMC4763993; doi:10.1371/journal.pone.0149926)

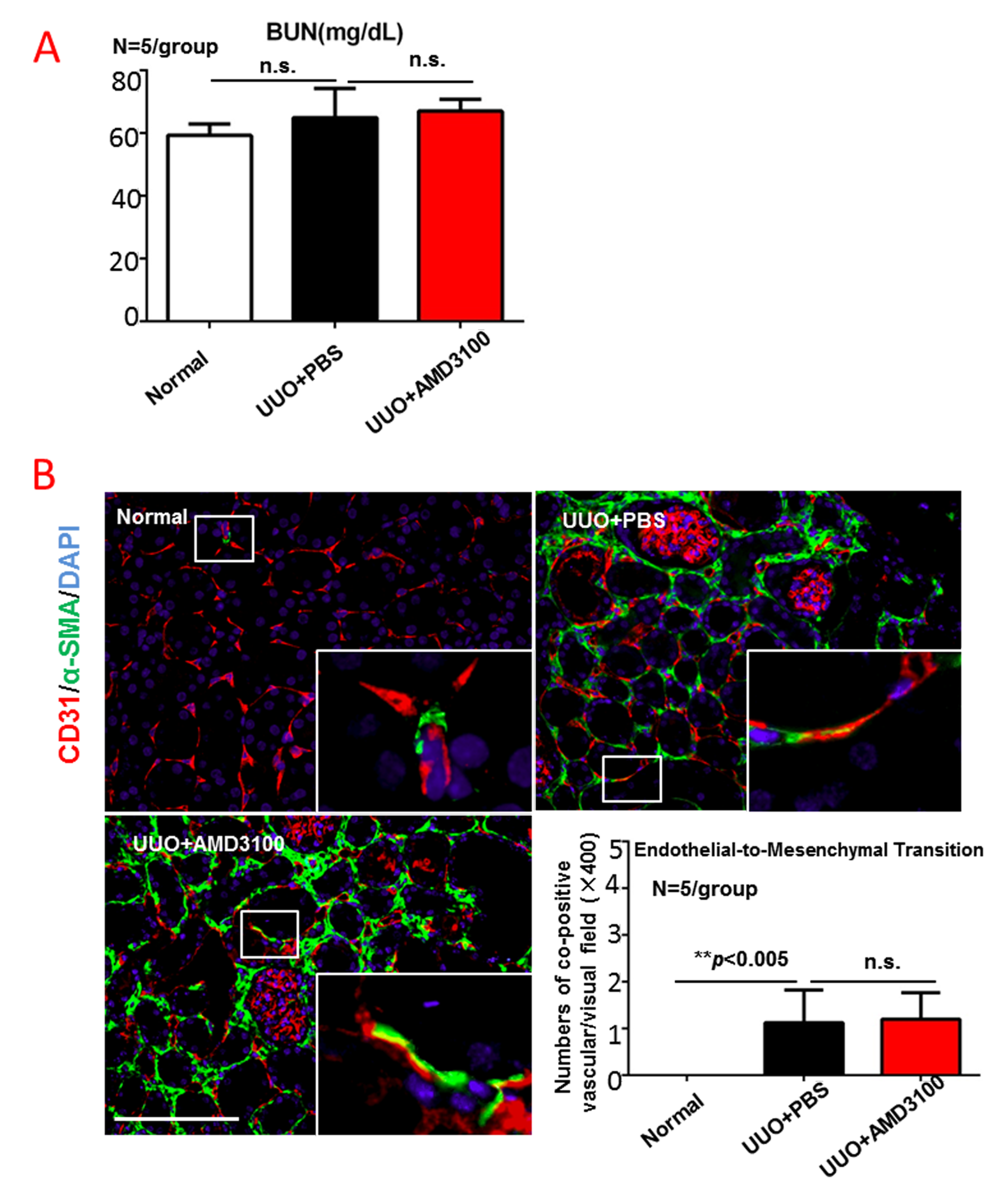

Supplement: S1 Fig — A. The effect of AMD3100 on renal function. Blood Urea Nitrogen (BUN) of three groups were detected and analyzed. B. The changes of Endothelial-to-Mesenchymal Transition after AMD3100 administration. Double staining of CD31 (red) and α-SMA (green) was detected by immunofluorescence, and the number of co-positive vascular (yellow area) was analyzed. Lower right corner pictures in IF figures were the higher magnification of inset areas with white border. (***p<0.001, **p<0.005, *p<0.05, n.s. means no significant statistical difference, scale = 100μm, magnification×400) (TIF) [file pone.0149926.s001.tif]
